# 351. Antibiotic Management Decisions and Use of a Multiplex PCR Panel for Pneumonia Diagnosis Among Critically Ill Patients with COVID-19

**DOI:** 10.1093/ofid/ofab466.552

**Published:** 2021-12-04

**Authors:** Neda Bionghi, Donald E Dietz, Jason Zucker, Jason Zucker, Simian Huang, Susan Whittier, Daniel A Green, Fann Wu, Magdalena Sobieszczyk, Deborah Theodore

**Affiliations:** 1 Columbia University Irving Medical Center and New York-Presbyterian Hospital, New York, New York; 2 Columbia University Irving Medical Center, New York, New York; 3 Columbia University Medical Center, New York, NY

## Abstract

**Background:**

Antibiotic use among patients with COVID-19 is common, exceeds the prevalence of probable bacterial co-infection, and promotes development of resistant organisms. Lack of diagnostic microbiological data may prolong empiric broad-spectrum therapy. Here we evaluate the use of the BioFire FilmArray pneumonia panel (PP), a novel rapid diagnostic test, and antibiotic decisions among intensive care unit (ICU) patients with COVID-19.

**Methods:**

We conducted a retrospective review of adult ICU patients admitted with COVID-19 between January 2020 and May 2021 at an academic medical center. ICU patients who underwent bronchoscopy/bronchoalveolar lavage (BAL) with PP (PP group) were matched by age (< 65 or ≥65), BMI (< 30 or ≥30), and BAL date (within 60 days) to ICU patients who did not undergo BAL (no-BAL group). PP patients were matched by age and BMI to ICU patients who underwent BAL without PP (no-PP group). Antibiotic use was compared between groups. Chi squared analysis, t-test, and ANOVA were used for comparisons as appropriate.

**Results:**

65 patients were included; the majority were male (65%), < 65 years (86%), and had BMI ≥30 (54%) (Table 1). Only 17 no-PP matches were identified for PP patients due to infrequent BALs. Similar proportion of patients in PP and no-PP groups had organisms identified from BAL (54% vs. 47%, p=0.65). Among PP patients with a detected organism, all (n=13) had subsequent changes in antibiotic regimen ≤72 hours after BAL; 10/13 (77%) had a change targeted to detected organism and 5/13 (39%) had antibiotic narrowing. Among PP patients with no detected organism, only 4/11 (36%) had antibiotic narrowing or maintenance off antibiotics. In all groups, average antibiotic use exceeded 70% of admission duration.

Table 1. Patient characteristics and antibiotic management. Abbreviations: BAL - bronchoalveolar lavage

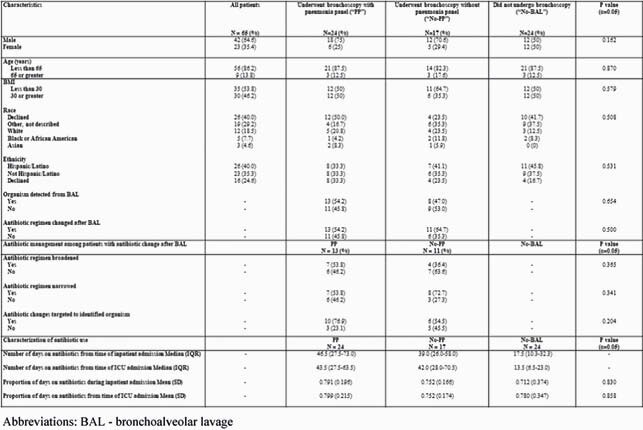

**Conclusion:**

Rapid, highly sensitive diagnostic tests have potential to guide clinical decisions and promote antibiotic stewardship among patients with severe viral pneumonia and suspected bacterial co-infection. In this descriptive analysis, antibiotic management did not differ significantly with use of PP. While most patients with detected organism on PP had targeted antibiotic changes, a negative PP did not appear to influence antibiotic narrowing. Larger studies and provider education are needed to evaluate potential of the PP for antibiotic stewardship.

**Disclosures:**

**Jason Zucker, MD, MS**, Nothing to disclose **Daniel A. Green, M.D.**, **BioFire** (Grant/Research Support, Scientific Research Study Investigator, Advisor or Review Panel member) **Deborah Theodore, MD**, **BioFire Diagnostics** (Other Financial or Material Support, Donation of testing materials to support investigator-initiated research)

